# Challenges in diagnosing thrombotic thrombocytopenic purpura

**DOI:** 10.1016/j.htct.2025.103842

**Published:** 2025-05-10

**Authors:** Jeremy W. Jacobs, Garrett S. Booth, Brian D. Adkins

**Affiliations:** a Pathology, Microbiology, & Immunology, Vanderbilt University, Nashville, TN, USA; b Pathology, University of Texas Southwestern Medical Center, Dallas, TX, USA

Thrombotic thrombocytopenic purpura (TTP) is a medical emergency necessitating rapid therapeutic intervention to prevent mortality. This rare hematologic condition is characterized by a deficiency in the ADAMTS13 enzyme (a disintegrin and metalloprotease with thrombospondin type motifs, member 13) [1]. ADAMTS13 is a metalloprotease which functions to cleave ultra-large von Willebrand factor (UL-VWF) multimers. Without ADAMTS13, these UL-VWF multimers bind to circulating platelets, resulting in the formation of microthrombi in arterioles and capillaries, leading to end-organ ischemia and hemolysis due to shearing of red blood cells (RBCs) as they transverse these microthrombi [2].

A deficiency in ADAMTS13 is most commonly due to autoantibodies which result in functional inhibition and/or accelerate the clearance of the ADAMTS13 protein from plasma (immune-mediated TTP - iTTP). However, in a minority of cases (<10 %), mutations in the *ADAMTS13* gene, cytogenetically located on chromosome 9q34.2, result in the inability to produce the ADAMTS13 protein or lead to the production of a dysfunctional enzyme [3]. In the case of abnormal or absent ADAMTS13 production secondary to genetic mutations, the condition is known as congenital TTP (cTTP) or Upshaw-Schulman syndrome.

In contemporary assays, ADAMTS13 activity is significantly reduced in patients with cTTP and iTTP. As such, it is not possible to differentiate these conditions by ADAMTS13 activity alone. However, this distinction is important, as the therapeutic, prophylactic, and prognostic characteristics differ. Moreover, there are instances where ADAMTS13 activity may be falsely low, or even undetectable, due to interferences in laboratory assays. Therefore, a nuanced discussion of the differences in cTTP and iTTP, and the assays used to diagnose the specific condition are warranted. We believe the importance of these nuances are particularly highlighted by a recent case of purported iTTP presented by Martins de Oliveira Filho et al. [4] In this report, the authors described a 50-year-old male with systemic lupus erythematous who was initially treated for immune thrombocytopenic purpura (antibody-mediated platelet destruction). The patient did not respond to corticosteroids, and further evaluation demonstrated evidence of thrombocytopenia and microangiopathic hemolytic anemia. The authors stated that “ADAMTS13 activity was undetectable, confirming a diagnosis of acquired thrombotic thrombocytopenic purpura”; while not incongruent with a diagnosis of iTTP, we believe that the information provided to the readers is insufficient to ‘confirm’ a diagnosis of iTTP. Further, we believe that this case illustrates the difficulties in confirming a diagnosis of iTTP, contributes to discrepancies in the literature, and precludes the ability to perform accurate epidemiological studies.

As mentioned above, both cTTP and iTTP are associated with low to undetectable (generally <10 %) ADAMTS13 activity. However, to definitively diagnose iTTP, an assessment for the presence of an autoantibody against ADAMTS13 should be performed. While not all patients with iTTP will have a detectable autoantibody, especially at initial presentation [5], the absence of such should evoke suspicion of cTTP wherein antibodies are absent [6]. As such, without the identification of an autoantibody, genetic testing should be performed to exclude mutations in the *ADAMTS13* gene. Given that the authors did not report an antibody, nor did they assess for genetic mutations, this case cannot be considered a ‘confirmed’ case of iTTP. While both iTTP and cTTP can present similarly with thrombocytopenia, hemolytic anemia, and end-organ ischemia, iTTP requires immunosuppression (corticosteroids, rituximab) to eliminate the inhibitor, plasma exchange to further reduce the acute effects of the inhibitor and UL-VWF and replenish the ADAMTS13 enzyme, and in many cases, an adjunct agent, caplacizumab, is used to further reduce microthrombi formation by inhibiting VWF-platelet binding [7] Conversely, cTTP usually only requires plasma infusion to replete the deficient ADAMTS13. Notably, recombinant ADAMTS13 has been approved in both Europe and the US, the use of which eliminates the risks inherent to exposure to donor plasma [8].

Secondly, it is important to mention the assays currently in use to evaluate ADAMTS13 activity, and how these assays may give a decreased result that is not due to an autoantibody or genetic mutation. At present, the most commonly used assays are based on either fluorescence resonance energy transfer (FRET) or enzyme linked immunosorbent assay (ELISA) technologies utilizing recombinant VWF substrates [9]. A variety of factors can interfere with these assays; for example, hyperlipemia, elevated plasma hemoglobin, or hyperbilirubinemia may interfere with fluorescence-based assays [10]. Free hemoglobin and bilirubin may also directly inhibit the ADAMTS13 enzyme, while other plasma proteases may interfere with VWF cleavage or degrade ADAMTS13 [10].

While iTTP has a greater prevalence than cTTP, if comprehensive evaluation is not undertaken (or reported), it is difficult to draw conclusions from the presented findings. Many patients present with low or inconclusive inhibitor results on initial presentation and must be followed to determine antibody status. Further, although many patients with cTTP present early in life, a subset do not develop overt disease until advanced age [11]. Given these considerations, in patients with suspected iTTP, it is important to thoroughly assess for not only ADAMTS13 activity but also autoantibodies; if the latter are not detected, further testing for cTTP is warranted. It is also important to understand the assay methodology for ADAMTMS13 activity, and the potential limitations and interferences, to ensure an accurate diagnosis.

## Contribution statement

**JWJ:** Reviewed the literature, drafted the manuscript, approved the final version. **GSB:** Revised the manuscript, approved the final version. **BDA:** Revised the literature, revised the manuscript, approved the final version.

## Conflicts of interest

The authors declare that they have no known competing financial interests or personal relationships that could have appeared to influence the work reported in this paper.

## References

[1] Joly B.S., Coppo P., Veyradier A. (2017). Thrombotic thrombocytopenic purpura. Blood.

[2] Moake J.L., Rudy C.K., Troll J.H., Weinstein M.J., Colannino N.M., Azocar J. (1982). Unusually large plasma factor VIII: von Willebrand factor multimers in chronic relapsing thrombotic thrombocytopenic purpura. N Engl J Med.

[3] Levy G.G., Nichols W.C., Lian E.C., Foroud T., McClintick J.N., McGee B.M. (2001). Mutations in a member of the ADAMTS gene family cause thrombotic thrombocytopenic purpura. Nature.

[4] Martins de Oliveira Filho C., Bode-Sojobi I., Lam B.D., Conrad S., Berry J., Carney B.J. (2024). Assessing treatment response in thrombotic thrombocytopenic purpura: beyond the platelet count. Hematol Transfus Cell Ther.

[5] Simon D., Leclercq M., Joly B., Veyradier A., Coppo P., Benhamou Y. (2024). Acquired thrombotic thrombocytopenic purpura without detectable anti-ADAMTS13 antibodies: a possible underlying autoimmune mechanism. Haematologica.

[6] Pérez-Rodríguez A., Lourés E., Rodríguez-Trillo Á., Costa-Pinto J., García-Rivero A., Batlle-López A. (2014). Inherited ADAMTS13 deficiency (Upshaw-Schulman syndrome): a short review. Thromb Res.

[7] Zheng X.L., Vesely S.K., Cataland S.R., Coppo P., Geldziler B., Iorio A. (2020). ISTH guidelines for treatment of thrombotic thrombocytopenic purpura. J Thromb Haemost.

[8] Scully M., Antun A., Cataland S.R., Coppo P., Dossier C., Biebuyck N. (2024). Recombinant ADAMTS13 in congenital thrombotic thrombocytopenic purpura. N Engl J Med.

[9] Favaloro E.J., Pasalic L., Henry B., Lippi G. (2021). Laboratory testing for ADAMTS13: utility for TTP diagnosis/exclusion and beyond. Am J Hematol.

[10] Peyvandi F., Palla R., Lotta L.A., Mackie I., Scully M.A., Machin S.J. (2010). ADAMTS-13 assays in thrombotic thrombocytopenic purpura. J Thromb Haemost.

[11] Alwan F., Vendramin C., Liesner R., Clark A., Lester W., Dutt T. (2019). Characterization and treatment of congenital thrombotic thrombocytopenic purpura. Blood.

